# Motivating healthcare professionals (nurses, nurse assistants, physicians) to integrate new practices for preventing healthcare-associated infections into the care continuum: turning Positive Deviance into positive norms

**DOI:** 10.1186/s12879-021-06215-3

**Published:** 2021-05-28

**Authors:** Anat Gesser-Edelsburg, Ricky Cohen, Adva Mir Halavi, Mina Zemach

**Affiliations:** 1 School of Public Health, University of Haifa, 199 Aba Khoushy Ave., Mount Carmel, 3498838 Haifa, Israel; 2 The Health and Risk Communication Research Center, University of Haifa, 199 Aba Khoushy Ave., Mount Carmel, 3498838 Haifa, Israel; 3 Midgam Consulting & Research Ltd. Derech Ben Gurion 13, 68181 Bnei Brak, Israel

**Keywords:** Healthcare-associated infections, Infections prevention practices, Hand hygiene, Positive Deviance approach, Norms, Qualitative study, Dissemination, Simulation, Healthcare professionals, Think Aloud, Recognition-Primed Decision, Video-Reflexive Ethnography

## Abstract

**Background:**

The literature examining healthcare-associated infections (HAI) points to two main problems in conforming to infection prevention and control (IPC) guidelines among healthcare professionals (HP). One is the discrepancy between HPs’ behavioral intentions and their implementation in practice. The other refers to how HPs maintain these practices after the intervention stage ends. The method proposed in this study seeks to address both these issues by using the Positive Peviance (PD) approach to focus on the dissemination stage of interventions. The study seeks to offer a method for disseminating 27 PD practices to 135 HPs, among them nurses, nurse assistants and physicians, so as to help them maintain IPC guidelines, offer feedback on the dissemination process and examine the impact of the dissemination stage on changes in their behavior.

**Methods:**

The theoretical model underlying this qualitative research was the Recognition-Primed dDecision (RPD) model, which we implemented in the field of healthcare-associated infections (HAIs). Moreover, we used the Discovery & Action Dialogue (DAD) and Think Aloud (TA) techniques to describe the methodological development of simulations for HPs. Feedback from the HP demonstrators underwent content analysis, while descriptive statistics were used to characterize behavioral changes.

**Results:**

HPs’ information processing regarding infection prevention shifts from peripheral/automatic processing to intuition and analytical/central processing, turning PD practices into positive norms. The HPs personally experienced finding a solution and made repeated corrections until they overcame the barriers. Most of the HPs (69.4%) reported that the practices were fully implemented, together with additional practices.

**Conclusions:**

Implementation of the dissemination stage indicates that in order for HPs to integrate and assimilate practices that are not in the official guidelines, merely observing simulations is not sufficient. Rather, each staff member must personally carry out the procedures.

## Background

Nosocomial infections, also known as health-care associated infections (HAIs) and hospital-acquired infections, constitute one of the most critical and investigated issues in public health worldwide [1, 2]. Despite accumulated knowledge and implementation of varied strategies in this field, hand hygiene (HH) compliance remains low, infection rates are high, and there is still a gap between recommendations and implementation [3, 4].

Numerous and diverse programs have been designed throughout the world to reduce the rate of HAIs, but there is still uncertainty regarding the effectiveness of each specific strategy relative to others or the effectiveness of a number of combined strategies [4–7]. A systematic review and network meta-analysis conducted in 2015 sought to evaluate the relative efficacy of the World Health Organization’s 2005 campaign (WHO-5) and of other interventions to promote HH among healthcare workers in hospital settings. The review found WHO-5 to be effective, yet additional interventions in conjunction with elements of the WHO campaign have the potential to lead to further improvements [8]. Strategies alone are clearly insufficient to achieve the goals. Indeed, behavior motivation factors must be incorporated in order to devise effective intervention programs [4, 8–12].

Even though HPs working in public settings are aware of the importance of maintaining infection prevention and control (IPC) guidelines, here too, as in other fields, there are still significant discrepancies between intentions and actual behaviors [13, 14]. Some of these discrepancies arise from what we termed “gray areas” in our previous study [15]. This term refers to the lack of solutions at different points along the care continuum (i.e., the range of medical procedures carried out during the course of a patient’s hospitalization(. At these gray areas, some HPs do not know what is required of them, leading to confusion, frustration, and various interpretations. Therefore, despite the importance of written guidelines, they cannot cover all the situations that may arise along the care continuum that may cause hospital infections to spread. The findings of our previous study indicate that written guidelines cannot be totally comprehensive, as they fail to account for the dynamic nature of the work and therefore are hard to translate into the work environment [15]. The pPositive dDeviance (PD) approach can help find solutions along the care continuum that are not contained in the official guidelines, thus narrowing the gap between intentions to maintain hygiene and actual behavior.

The PD approach is an innovative behavioral approach to solving complex problems (e.g., HP compliance in maintaining IPC guidelines). The approach addresses two key parameters that emerge from the literature: 1) the need to find solutions from within the community’s existing resources, and 2) the need to empower HPs by identifying individuals who behave in exceptionally positive ways. These individuals (i.e., people within the community whose behavior instills change) serve as role models by virtue of the fact they have developed successful solutions and strategies for dealing with problems without resorting to additional resources unavailable to fellow members of their community [16]. In the current study, these individuals are nurses and physicians who have developed unique and successful solutions and strategies. Since PD is a community-based approach that operates from the bottom up (from members of the community to management), it takes into account all the “situational factors” (i.e., factors in the social and physical environment that block or facilitate processes of change) associated with the organizational culture of medical teams in their daily reality. By identifying positive behaviors, the approach is able to offer sustainable solutions to many situations.

Implementing the PD approach has the potential to reduce the gap between intentions and actual behavior reported in the literature, increase the rates of HP compliance with infection prevention rules, and reduce infection and mortality rates [15].

The PD methodology consists of four basic steps carried out by members of a community.

Step 1: Identify “positive deviants,” i.e., individuals who consistently demonstrate exceptionally high performance in an area of interest.

Step 2: Study these individuals in depth using qualitative methods to generate hypotheses about practices that enable organizations to achieve top performance.

Step 3: Test hypotheses statistically in larger representative samples of individuals.

Step 4: Work in partnership with key stakeholders, including potential adopters, to disseminate the evidence about newly characterized best practices.

This article focuses on Step 4 of the PD approach, namely the dissemination of new practices. Disseminating practices among other HPs depends on how they assimilate their new knowledge. The process of internalization and assimilation can be explained by the Recognition-Primed Decision (RPD) model [17, 18], which demonstrates how information is processed. The RPD model describes how professionals use their experience to make rapid decisions in time-pressured settings under conditions of uncertainty [17, 19, 20]. In accordance with this model, we seek to demonstrate how HPs make decisions in complex situations related to IPC guidelines.

The RPD model encompasses two steps individuals must adopt in making decisions. First, they must recognize which course of action makes sense. After that, they must evaluate this course of action by imagining whether the actions resulting from the decision make sense. In this decision-making process, experience plays a major role.

HPs must move from one complex task to another. In such a reality, identifying specific situations that require conforming to IPC guidelines presents a challenge. The literature offers a number of techniques that can help HPs complete their behavioral intentions of maintaining IPC guidelines. For example, the study by Fuller et al. [21] attempted to address the complex ongoing problems faced by HPs by creating hints to help them remember hygiene procedures. The study suggests that future interventions should be developed in cooperation with HPs to build “if-then” programs: “If X happens then I will do Y ….” This technique, which can help HPs shift from one task to another and choose the best solution, is implemented through the Think Aloud (TA) method, a research method used to study cognition that is considered the optimal method for capturing thought processes, particularly for problem-solving. In the current study, TA was applied to the implementation of IPC guidelines.

While using TA, individuals verbalize how they are using available information to generate a solution to a problem. Unlike other techniques for gathering verbal data, TA entails no interruptions or suggestive prompts or questions. The TA participant is encouraged to concentrate on the tasks being performed while verbalizing a continuous stream of thoughts and avoiding interpretation or explanations [22]. TA can transform the thought processes of expert clinicians, which are usually automatic and implicit, into explicit and concrete explanations. TA reveals steps in the reasoning process and makes explicit how decisions are made. It emphasizes the process of making a diagnosis, rather than just focusing on the diagnosis [6].

The TA process can help nurse educators teach nursing students how to identify and correct reasoning that is not up to par and show them scenarios that might arise during their clinical assignments. The educators can use the TA approach to help promote clinical reasoning strategies, such as hypothesizing, judging, and inductive and deductive logic. Nursing students can be evaluated on how they comprehend and verbalize what is taught in classroom lectures and how they connect scientific facts with health-related outcomes in order to identify problems and concerns. Not only must nurses be able to use diagnostic reasoning, they must also be flexible, knowledgeable, and capable of reflecting on approaches to clinical work while developing reasoning skills.

Other studies suggest that physicians undergo a similar cognitive process in making clinical decisions [23–25]. Chan et al. (2020) demonstrated that emergency physicians interviewed according to TA protocols engaged in iterative and dynamic decision-making processes that changed throughout their encounters with patients, in accordance with multiple contextual features [23]. Thus, the TA method helps connect the clinical experience to an array of strategies that affect patient-centered healthcare outcomes [26].

The current study is based on a large-scale study conducted in 2017–2019 in three Israeli hospitals and focuses on the information dissemination stage of PD practices (Stage 4) in reducing healthcare-associated infections (HAIs). To the best our knowledge, no studies have investigated how HPs assimilate new behavioral practices demonstrated by PDs who maintain IPC guidelines. We also found nothing in the literature examining the work of HPs who use RPD to prevent acquired infections. This article describes an application of the model and suggests methodological developments in the context of maintaining IPC guidelines.

The first goal of the research is to offer a method for disseminating PD practices for maintaining IPC guidelines to other HPs. The method is based on a number of tools that have been used in other fields, as specified below. The second research goal is to examine feedback from the demonstrators regarding the proposed method. The third goal is to examine the impact of PD intervention on HPs’ reported behavioral changes in maintaining IPC guidelines.

## Methods

### Research design

This article focuses on Step 4 of the PD approach, namely the dissemination of practices. A necessary condition for disseminating practices among other HPs depends on how they assimilate their new knowledge. We used the TA technique to explain the learning process and the RPD model to illustrate how the participants process the information.

**Fig. 1:**
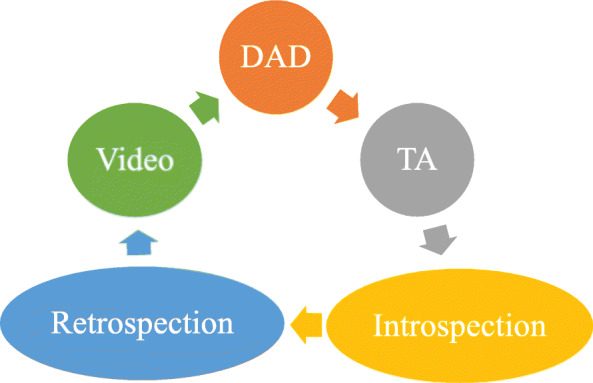
Stages in the research process. Specifications of model’s different stages are presented in the research process

RPD served as the theoretical model underlying this research. The model focuses on decision-making under circumstances of stress and intense conditions. Thus it is suitable for the medical field, where HPs are required to find effective solutions. In this study we outlined the methodology of the theoretical model by using various tools that facilitate its use and accommodated it to the issue of reducing HAIs and maintaining IPC guidelines. The research model illustrated in Fig. 1 demonstrates the correction and repetition process, until the ultimate solution for the gray areas along the care continuum in hygiene maintenance is found.

We used a technique that integrated TA and retrospection, wherein TA protocols or behavioral observations during a session are used to obtain a retrospective protocol of any pauses in the TA session or fragments that were incomprehensible, incomplete, or odd and introspective. The TA method then uses more direct data for validation [22] and is supplemented by DAD. We added the element of dissemination by video recording via smartphones. The entire demonstration process was filmed, including repetitions and corrections made until the final product was obtained. We assumed that the video recording influenced the demonstrator’s assimilation process.

### Study population

The study was conducted at three hospitals in Israel: Hadassah Ein Kerem Medical Center, Bnei Zion Medical Center, and Rambam Medical Center. Five departments/units participated: one Medical Intensive Care Unit (MICU), two internal medicine departments and two orthopedic departments.

The study included 135 HPs who participated in the dissemination stage; 27 of them served as the demonstrators (Table 1). Each of the demonstrators performed a single behavioral practice/procedure in the dissemination stage and documented it by video.

**Table 1:** The healthcare professionals sociodemographic characteristics

Sociodemographic characteristics	Category	Total HPs (***n*** = 135) n (%)	Demonstrators (***n*** = 27) n (%)
**Gender**	Male	39 (29)	9 (33)
	Female	96 (71)	18 (67)
**Age (years)**	Mean (range)	38 (22–65)	38 (22–65)
**Ethnicity**	Jewish	82 (61)	15 (56)
	Arab	53 (39)	12 (44)
**Sector**	Nurse	115 (85)	21 (78)
	Nurse assistant	12 (9)	3 (11)
	Physicians	8 (6)	3 (11)
**Seniority (years)**	Mean (range)	11 (0.5–38)	13 (1.5–38)
**Hospital/Department**	Hospital 1/ MICU	37 (27)	7 (26)
	Hospital 2 / Internal Medicine	26 (19)	5 (19)
	Hospital 2 / Orthopedics	20 (15)	6 (22)
	Hospital 3 / Internal Medicine	31 (23)	3 (11)
	Hospital 3 / Orthopedics	21 (165)	6 (22)

### The research process

As noted, this study is part of a larger study conducted from 2017 to 2019 and is based on our previous published findings. Some of the PD practices discussed in this article came up in our earlier work (see details below), while three of them (Practices 1, 10 and 11) are mentioned here for the first time.

The 27 practices documented by video included 13 of the PD behavioral practices along the care continuum: (1) changing a surgical dressing; (2) removal of protective clothing when leaving an isolation room, and carrying out hand hygiene [15]; (3) taking a blood sample [15]; (4) sending blood samples to the laboratory [15]; (5) inserting a central line [27]; (6) washing a patient in bed [15]; (7) sterilizing a stethoscope [15]; (8) cleaning a patient’s unit and surroundings [15]; (9) taking a patient’s urine sample with a urine catheter and sending it to the laboratory [15]; (10) cleaning the nursing station; (11) mixing IV meds and bringing them to the patient; (12) replenishing disposable equipment in a patient’s room; and (13) instructing patients and families on how to maintain hygiene in the hospital [28].

Three of the 27 practices documented by video require maintaining sterility and using sterile equipment in addition to maintaining hygiene: (1) changing a dressing attached to a central line; (2) inserting a urine catheter; and (3) suctioning a respirated patient.

#### Building the setting

The procedures were performed in the hospital in their natural setting. In most cases, the facilitator/demonstrator was a medical staff member specializing in the procedure, while another HP who was a member of the research team filmed and documented the procedure. In some cases the facilitator guided, filmed and documented the procedure using a smartphone. Each HP worked on one of the 27 practices, and each of them conformed to the integrated three-stage model, as follows:

*In the first stage,* we asked each of the 27 HP demonstrators to demonstrate one procedure/practice. Prior to the demonstration, the PD facilitator explained the approach and performed the procedure. During the demonstration and filming, the facilitator applied the DAD method, asking the demonstrator to find solutions to situations for which there are no clear guidelines. In accordance with the TA method, the demonstrators were told to speak out loud about their problems and deliberations, while explaining their actions step-by-step. At any point when demonstrators stopped, the facilitator asked why they stopped and how they thought to continue. It is important to note that every time the demonstrators criticized themselves (e.g., by stating they had acted in a way that did not maintain a hygienic/sterile environment), they repeated the action again. In effect, they repeated the action again and again several times, making corrections until finding the optimal solution/frame.

*In the second stage*, which took place about 1 month after filming the procedure, we showed the demonstrators the videos again and interviewed them about the influence and effectiveness of the procedure, using the TA introspection prompting technique. We chose a time period of 1 month to allow the demonstrators to gain perspective about the process.

*In the third stage,* we asked all HP observers to rate each practice by indicating the extent to which it had changed their behavior and motivated them to implement the practice, on the following scale: 1 - I did not implement it; 2 - I implemented it partially; 3 - I implemented it fully; 4 - I implemented it fully and added my own practices.

### Analysis

The materials underwent content analysis as follows:

*In the first stage* we analyzed the transcripts of the texts spoken during the filming, including the correction stages, until the speakers found solutions during the medical procedure. We then constructed the following index of criteria for analyzing the processes: (1) identifying the gray area on the continuum of actions (defining the problem); (2) corrections made by the demonstrator; (3) quotes from the demonstrator’s correction process; and (4) thematic analysis of the demonstrator’s evaluation of the process.

*The second stage* entailed thematic analysis [29] of the 27 personal interviews held with the demonstrators that focused on the effectiveness of the method and its influence on them.

The thematic analysis was conducted independently by two researchers to ensure reliability. These researchers identified relevant themes and sub-themes concerning participation in the research model. The analysis began by reading through the transcripts for general and potential meanings. Then, each researcher created an initial coding structure based on the descriptive coding by coding units of text as themes and labeling them with a phrase related to the participant’s account. Next, the researchers conducted a joint analysis that entailed consolidating and prioritizing the independently identified themes, yielding the initial thematic framework of the analysis.

*In the third stage,* the behavioral changes reported by the HPs were analyzed using descriptive statistics. We pooled all practices identified at the three hospitals and assessed the proportion of participants who rated each of the implementation levels.

### Validity and credibility of the procedure

The validity of each action performed by the demonstrator to prevent contamination was scientifically approved by the two HPs present at the event. In addition, an external HP from the infection control unit viewed the videos and approved the final results.

The interviews and videos were analyzed by three different researchers. These analyses were then compared, and agreed-up themes were identified. In the very few cases where there was disagreement, we went back to the transcripts and the videos.

## Results

Table 2 shows three examples of sterile procedures (inserting a urine catheter, changing a dressing for a central line, suctioning a respirated patient) performed by the demonstrators. For each example, the table indicates the gray areas (problem definition), solutions/corrections proposed by the demonstrators, quotes from the demonstrator’s statements during the correction process, and demonstrator feedback (in the form of retrospection about the effect of the demonstration). These elements describe how PD practices are disseminated to other HPs in order to maintain IPC guidelines.

**Table 2:** Anaysis of the demonstration of sterile procedures including process evaluation. The demonstration is performed on the basis of the order of actions with a view to reducing contamination

Practice	Presentation of the gray area on the action continuum (definition of the problem)	The correction as performed by the demonstrator	Quotes from the correction process by the demonstrator	Feedback on the process – retrospection by the demonstrator on the effect of the demo
**Inserting a urine catheter (sterile practice)**	1. Preparing the equipment and placing it on the patient’s bed. In this situation the equipment can scatter and fall off of the patient’s bed, and can also be outside of the nurse’s field of vision. This can interrupt the insertion procedure and break sterility.	Preparing equipment in advance by order of use, placing it on the wagon in an accessible place for the nurse.	“When I prepare all of the equipment in advance on the wagon, I see everything with my eyes and I arrange it in the order of what I’m going to need for each stage. Then I don’t forget anything and have to run in the middle of the procedure to bring something and then take my gloves off and sanitize my hands again.”	**General evaluation of the model:** “Since I demonstrated, I’ve been even stricter. If they could show us one procedure at a time in the form of a video, that would be amazing.” **Filling out the gray area:** For instance, we emphasized all kinds of things that aren’t in the guidelines, such as putting your name tag in your pocket before you begin, and bringing a garbage bag, because it helps, and every little thing like that can reduce contamination.” **Raising difficulties on the care continuum: “**A lot of people slip, for example, when they get to the part of spreading the patient’s legs. It’s difficult. And there are lots of moments where you can break sterility there. We learned how to talk to each other about the preparation you have to do.” **The contribution of TA:** “We plan what we do and say it out loud one step at a time, and I say what I’m going to do step-by-step.” **Processing information by the analytical route:** “These videos completely refresh what we do all the time, and sometimes there are little tips that help in the places we miss and make mistakes from stress and pressure...” **The contribution of filming: “**When you watch such a video, it’s not like when they hand you a page and tell you these are the guidelines, read about how you insert a urine catheter. When you see the video and then you approach a patient, you remember what you saw.”
		Correct and efficient planning of the insertion procedure according to the number of staff performing the procedure	“If there are two staff members it makes the work easier and we divide it between us, with me being the nurse that performs the insertion while the other nurse is responsible for the equipment and handing it to me sterilely in order of the stages.”	
	2. The physical preparation of the bed and the patient (making the bed, removing the blanket, changing position, raising gown and spreading patient’s legs) when the equipment is at the patient’s feet rather than on a dedicated wagon, it might break the sterility of all of the equipment.	Instructing the patient, preparing the equipment in advance on the wagon, physical preparation of the patient and their surroundings.	“The matter of preparing the patient is critical and facilitates all of the subsequent actions. When you have easy access to the equipment and to the insertion site, the chance of breaking sterility are low.”	
	3. Bending over to the patient in order to begin the procedure and contact of staffer’s name tag with the patient’s surrounding (blanket, open abscesses on the patient’s body). The name tag can serve as a vector for transmitting contamination, in addition to potentially breaking sterility in the course of the insertion.	Putting the staffer’s name tag in their pocket – to prevent contact with the patient’s surrounding and the patient.	“When I bend over toward the patient, my name tag swings towards the patient along with me, and it can break the sterility at the insertion site.”	
	4. Disposing of the waste on the patient’s bed until the end of the insertion. The waste can scatter, fall off the bed, and be a vector for transmitting contamination.	Preparing a garbage bag in advance for equipment waste and laying it out on the lower part of the patient’s bed, with the nurse having easy access to deposit waste into it.	“In the very first stages, after I explain to the patient the action I’m going to perform, I put an open bag at the foot of the bed near the insertion site so I have easy access to dispose of the waste that piles up during the procedure directly into the bag and not on the bed itself.”	
**Changing dressing for central line** **(sterile practice)**	1. Planning the sterile field in the patient’s surroundings, which is crowded and surrounded by a curtain and does not enable a wide and safe range of movement. This fact reduces the nurse’s access and makes it very difficult for the nurse to lay out the sterile field and keep it sterile.	Preparing equipment in advance on the wagon by order of use and thinking about preventing contamination when laying out the sterile field. This fact helps prevent unnecessary movements in the workspace.	“We always try to prepare the equipment in advance, but you don’t really think about where you place each thing and how much room you leave on the wagon to spread out the sterile field.” “I prefer to prepare the equipment in advance by the order I will need to use it, one thing after another, so that I don’t forget anything.”	**Processing information by the analytical route.** “Sometimes we forget very essential things about the actions we perform and then we have to stop and refilm the video.” **Filling out the gray area.** “I learned so much from the process: it highlights our daily activities and flashes a red light about all kinds of little situations on the continuum that we sometimes forget, that have to do with hygiene maintenance.” **The contribution of filming.** “To see a video today is more practical and interesting than reading a boring procedure.” **The contribution of diffusion. “**And I can already see that after it was passed on to staff members through staff meetings, people are starting to implement it.“
	2. Direct transition from a nonsterile action to a sterile action. This situation creates confusion for the nurse because the sterile field was already prepared but she has to perform the act of removing the existing dressing with regular gloves, before she puts on sterile gloves in order to apply the new dressing.	After preparing the sterile field, the nurse divides the practice into two parts. She calls the first part the “non-sterile” stage and declares it out loud, while removing the existing dressing with regular gloves. Then she begins the second part, which she calls the “sterile” stage, in which she performs HH and puts on sterile gloves.	“After I prepare everything, I look at the field and make sure I didn’t forget anything, and then I perform hand hygiene, put on regular gloves, and perform the nonsterile part, while declaring out loud and removing the existing dressing, and only then, before I sterilize the catheter entry site, I become sterile.”	
**Performing suction on a respirated patient**	1. Preparing equipment in advance on the patient’s locker and using sterile water directly out of the sterile water bottle. Performing HH and putting on gloves.	Preparing the equipment in advance on the patient’s locker: Pouring sterile water into a disposable cup for washing the suction system at the end of the procedure.	“We hurry and most of the time we draw the water directly from the sterile water bottle, and then all of the water in the bottle becomes contaminated, and the bottle stays there until next time.”	**The contribution of diffusion.** “Actually, since we made this video it was sent to everyone, and I noticed that me and my staff have been stricter. Especially, the order we decided on became a habit.” **The contribution of repetition until reaching the final outcome.** “These stops are good (stopping the filming every time there is a breach) because they let you look close up in real time at what’s happening now, what’s wrong, and to think and correct the action.” **Evaluation of the model:** “Today I think it would be good to do this for every action. There’s no question that after this process we developed a practice that’s more correct and effective to reduce the risk of contamination. It’s really a unique project, I’ve never been exposed to a project like this at a hospital before.” **Assimilating the process. “**In retrospect, after you see the final film once or twice, it becomes a habit, you do it automatically and it becomes easier.”
		Preparing the sterile glove and pumping catheter above the patient’s blanket in a convenient site to guarantee access to equipment and prevent its falling.	“If I perform the procedure alone and there’s nobody to hand me the equipment, it’s important for me to put it in a place where it will be easy for me to take it and use it... After I disconnect the patient from the respirator, I carefully put on the sterile glove with the hand with which I’m performing the procedure, and then pull the cover over like this (holding under the armpit) and immediately grasp the catheter with my sterile hand.”	
	2. It’s difficult to maintain the sterility of the hand with the sterile glove and the catheter right before the insertion procedure.	When preforming the action as a single staff member – disconnecting the patient from the respirator, putting on the sterile glove, and removing the catheter wrapper with the armpit to prevent direct contact between the catheter and the opposite hand (which is not sterile).		
	3. Drawing sterile water directly from the sterile water bottle. In this case, inserting the used catheter into the bottle contaminates all of the water in the bottle and provides fertile ground for microbe proliferation. Usually staff members do not use a disposable cup but draw directly out of the bottle, thinking that this is an action that is performed at the end and the water is not inserted directly into the patient.	Drawing sterile water from water prepared in advance in a disposable cup.	“When I finish the suction, I take the catheter out gently and roll it directly into the internal part of the glove so as not to contaminate the whole surroundings.”	
	4. Placing the catheter on the bed and collecting it with the rest of the equipment and throwing it in the garbage. The catheter contains discharges from the patient and can contaminate the environment.	Rolling the catheter and inserting it into the used glove and collecting the rest of the waste and throwing it into the garbage.		

Table 3 outlines the thematic analysis of the 27 demonstrators’ retrospective feedback.

**Table 3:** Thematic analysis of the practice demonstrations by healthcare workers documented in the retrospection videos

Central theme	Quotes
**1. General evaluation of the model**	“As far as I’m concerned, the use of the videos came just in time. It empowered the staff and validated tips that are not present in the regular guidelines.” “Actually, since we made this video it went (viral) to everyone, and I noticed that me and my unit staff have been stricter. Especially, the order we decided on became a habit.” “It’s amazing to see how with simple actions are when the staff member explains what they are doing, it makes it applicable and accessible and I suggest that we disseminate these films to the other departments, so that the teaching will continue to spread.” “To see a video today is more practical and interesting than reading a boring procedure and I can already see that people are starting to implement it during their shifts.”
**2. Data processing by the analytical route** (Effective assimilation of the practice)	“When you see in the video how doctors peform, and you understand the risk of introducing contamination through the central-line, it is definitely an assimilation process that probably really doesn’t happen in any other way, and I can tell you that even though we have great relations with the staff, it’s been years since we’ve seen such an assimilation process, and it was received in a way that you don’t see using other techniques.” “We asked an orderly to demonstrate on a doll how she washes the patient, with all the knowledge and tips she brings from working in the field... Then other staff members demonstrated again with her, and you can see that they really assimilated the actions and it’s amazing.” “When we made the video it got me focused 100% and I gave it my best, even though these are things I do every day. Definitely there are things that were added in the video that I would not have necessarily done before, but since we made the video now I imitate the way I did it in the video.”
**3. The contribution of filming:** The contribution of filming is that it: a. Promotes the assimilation of practices more effectively than reading a procedure b. Actively involves the staff c. Empowers and increases sense of professionalism	“It’s amazing to see how this process serves as a mirror to each other of how the staff approaches things. At the end you could see the shift in how people assimilated and implemented things and talked about it.” “It was amazing to see in the process how there are things everybody is used to doing, the ‘norms,’ and then there are the exceptional tips that came up during the research, and how the rest of the staff started to implement them because of the videos.” “Even though we perform this procedure every day, the video gave us a kind of ‘back up,’ it also gives you a good feeling to keep doing it, and since then I’ve had more confidence that I’m doing the right thing.” “Merely documenting the practice in a video got me to be more organized... To make sure that I have the full and tidy equipment in advance and that I will not have to stop in the middle and ask somebody for help... It makes you feel more professional, and when you’re being filmed it forces you to meet the standards, especially if you want other people to learn from you. Simply by filming, it gives you the feeling that it’s your mission to pass the information on to younger nurses and students and that this will serve us going forward.”
**4. Bringing up difficulties on the care continuum nd filling out the gray area**	“We saw that precisely in the simple and basic practices we perform dozens of times a shift, like a peripheral venous catheter, drug dilution – there are so many gaps, and filming and watching the videos created a photographic memory that is much stronger than skimming over a written procedure.” “It made things clearer in my mind, when you talk about something, I used to do it spontaneously, and after the film I started to think about each and every stage, and to separate the stages, the disinfection stage, the stage of carrying the medication to the patient, I didn’t think about the process itself in this way before, the little details. Such as how to carry a medication from the medication room to the patient’s room. We used to say that you have to prepare the drugs sterilely, and the thing is, there’s a correct way to transfer the drug sterilely and not just to take the bag and hang it by the patient.” “These videos completely refresh what we do all the time, and sometimes there are little tips that help in the places we miss and where we do things wrong out of pressure...” “During your daily work sometimes you miss things, and when we made the video it got me to focus 100% and give it my best, even though these are things I do daily. For instance, before, when I changed the patient’s dressing and brought a garbage bag for the waste, I would put it on the floor. Today I say to myself, why on the floor? Why not hang it close to the patient?” “There are a lot of nurses who slip on the issue of decontamination, moving from inward to outwards. Everybody knows you’re supposed to do it that way but they don’t do it, and demonstrating the procedure with a short video is ideal.”
**5. The contribution of TA**	“I think the fact that you talk and explain while performing the actions makes you aware of everything you do, of every small details. It clarifies all kinds of points in your mind, things you already know.” “Saying out loud what I’m about to do gives me of a sense of confidence and it is sort of a control of myself because it makes you think about the next stages and not just work on auto pilot and it really helps.” “I think talking out loud... It also helps to share with people, to get ideas... For instance, the demonstration of taking blood, we didn’t do it like that before but now we do it like in the video.” “Because of the video we learnt to talk to each other about how to prepare what we need to do, to plan what we do, and say it out loud one step at a time, and say what we are going to do in the next step. Now our actions are synchronized and coordinated with each other.”

The following is a brief explanation of the five key themes emerging from the demonstrators’ feedback:

1. **General evaluation of the model of the proposed method:** The demonstrators emphasized the applicability and accessibility of the model to other HPs in raising awareness.
2. **HP information processing by the analytical route:** The demonstrators emphasized the quick and effective assimilation of practices facilitated by using the model.
3. **Contribution of filming**: (1) promotes assimilation of practices more effectively than merely reading a procedure; (2) actively involves the staff; (3) empowers and enhances sense of professionalism.
4. **Bringing up difficulties along the care continuum and filling in the gray area**: The demonstrators noted that during the practice demo, they were able to address and find solutions to the problems that arose during the demo, whereas in day-to-day work they failed to notice “problems.”
5. **Contribution of TA:** The TA technique in which demonstrators are asked to speak aloud caused them to plan and think about every step they took during the demo and encouraged a sense of security and sharing among the observers.

**Fig. 2:**
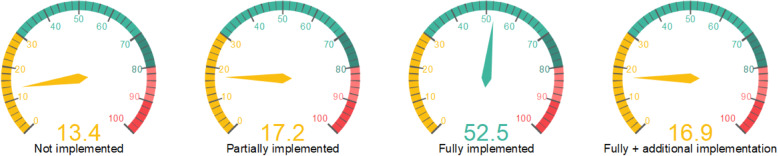
Levels of PDs practices implementation by all HPs (%). Four levels of the distribution of the overall PD practice’s implementation ranked by all the HPs after participating in the demonstration simulation are shown

Figure 2 shows the distribution (%) of the overall implementation of PD practices as ranked by all the HPs after participating in the demonstration simulation. According to Fig. 2, most of the HPs (69.4%) rated the practices as fully implemented and fully implemented + additional practices.

## Discussion

The model proposed in the present study overcomes two discrepancies noted in the introduction regarding the maintenance of IPC guidelines: 1) the gap between intentions and practical implementation, and 2) the problem of maintaining behaviors after the intervention stage, namely assimilation and practical maintenance without external oversight [30]. As we noted, the literature focuses on intervention rather than on maintenance. The methodological model proposed in our study suggests a method to overcome this issue by applying the RPD theoretical model to the field of infection control [17]. In the dissemination process examined in our study, the demonstrators were exposed to barriers and defects in the care procedure and overcame these while receiving facilitator support.

Reay and Rankin [31] claim that, in contrast to other abstract theories, the RPD model takes into account dynamic and stressful situations such as those found in the medical field. In RPD Variation 3, a person knowledgeable about the situation must choose the optimal course of action. The decision-maker engages in mental simulation that entails testing different scenarios using the “if … then …” format. After testing a number of scenarios, the decision-maker chooses a course of action that appears appropriate to the goals and priorities of the situation. Since the RPD model is predicated on time constraints, the decision-maker chooses the first course of action that appears appropriate to the situation. Our proposed methodological model implements RPD Variation 3: Each of the demonstrators must undergo a process in which the topic of maintaining a hygienic environment moves from the peripheral route to the central route, in accordance with the Elaboration lLikelihood mModel [32].

Unlike studies that examine analytical versus intuitive decision-making [33], we chose the RPD model as a path toward combining both. Identifying patterns requires intuition, whereas analytical reasoning is needed for mentally simulating options. Klein [19] argues that both are necessary because intuition alone may lead to faulty options and using only analytical reasoning would be too slow in time-pressured situations.

In other words, our findings show that in processing information concerning infection prevention, HPs move from peripheral/automatic processing to intuitive and analytical/central processing. The HPs’ personal experience in finding a solution while verbalizing their considerations using the TA technique and repeatedly correcting themselves until they overcome the barriers encountered shifted the issue of infection prevention from the automatic peripheral to the central analytical route. This increases the likelihood that the demonstrators will continue to maintain IPC guidelines while performing this procedure in the future. In addition, documenting the procedures by videotaping with smartphones, a readily available device, raises the likelihood that other HPs will watch the filmed procedures. Indeed, this is what happened based on our interviews with the demonstrators and the other HPs who observed the simulation. The interviewees reported that they incorporated the demonstrated practice into their routine work, and 69.4% of them reported that they changed their behavior in line with the PD intervention.

In addition, note that a growing body of research has been conducted using Video-Reflexive Ethnography (VRE) [34, 35] to assist healthcare workers understand IPC practices and devise solutions to these. This approach is similar to the dissemination method we described above in that it uses video for documenting HPs’ IPC practices. Yet unlike our method, VRE documents practices that are demonstrated without any interference. The HPs then view the video documentation in reflective focus group sessions where they discuss how to find solutions that are adapted to the changing reality.

Moreover, while the literature describes how the PD method identifies exceptional people who find solutions to complex situations along the care continuum and disseminate their practices to the rest of the staff members, exactly how this transfer process takes place was not discussed. The method introduced in the current study points out the different stages in the transformation process, thus motivating other HPs to be as creative as the PDs and transforming the desirable behavior from a deviance to a norm. This is because the HPs who participated in the PD study not only implemented the practice, but added to it during the intervention process, and some even offered their own tips. Therefore, the proposed model raises the likelihood that HPs will not only maintain hygiene in the practices they watched or experienced, but will also apply the approach and the skills they acquired to other procedures along the care continuum. Follow-up studies in the future can examine whether the HPs who participated in the demonstration in the dissemination stage implemented the approach in their ongoing work.

### Limitations

The research limitation is that the study examined and evaluated the PD intervention in specific hospitals. Therefore, further research is needed to explore the impact of this methodological model at other hospitals. In addition, beyond the research and evaluation stage, the assimilation and maintenance of the behavior change should be studied and evaluated over time.

## Conclusions

The implementation of the dissemination stage indicates that in order for HPs to integrate and assimilate practices that are not in the official guidelines, they must carry out the procedures themselves. It is important to note that implementation of this methodology does not require special resources on the part of the hospital system. The procedures can be demonstrated and practiced at different times in the hospital wards. Smartphones can be used for documentation and filming, and all the instructions can be delivered by the HPs themselves.

## Data Availability

Requests for more detailed information regarding the study can be addressed to the corresponding author.

## Abbreviations

IPC: Infection prevention and control
HAI: Healthcare-associated infections
HH: Hand hygiene
ICU: Intensive care unit
PD: Positive Deviance
RPD: Recognition-Primed Decision
TA: Think Aloud
WHO: World Health Organization
HP: Healthcare professional
DAD: Discovery & Action Dialogue
VRE: Video-Reflexive Ethnography
